# Supplementary material for: TAT-Modified ω-Conotoxin MVIIA for Crossing the Blood-Brain Barrier
Source: Mar Drugs. 2019 May 12;17(5):286. doi: 10.3390/md17050286 (PMC6562543; doi:10.3390/md17050286)
Supplement: Supplementary file 1 [file marinedrugs-17-00286-s001.pdf]

Article

TAT-Modified ω-Conotoxin MVIIA for Crossing the Blood-Brain Barrier

Shuo Yu[2,†], Yumeng Li[3,†], Jinqin Chen[2,†], Yue Zhang[2], Xinling Tao[3], Qiuyun Dai[2], Yutian Wang[4], Shupeng Li[3,5,6*] and Mingxin Dong[1,2*]

1 Institute of Neuroregeneration & Neurorehabilitation, Qingdao University, 308 Ningxia Street, Qingdao 266021, China
2 Beijing Institute of Biotechnology, Beijing 100071, China; o_yys@163.com (S.Y.); chenjq0210@163.com (J.C.); zy570524967@163.com (Y.Z.); daiqy@mail.bmi.ac.cn (Q.D.)
3 State Key Laboratory of Oncogenomics, School of Chemical Biology and Biotechnology, Peking University Shenzhen Graduate School, Shenzhen 518055, China; wendyleeym@163.com(Y.L.); taoxinling_hqu@163.com (X.T.)
4 Djavad Mowafaghian Centre for Brain Health and Department of Medicine, University of British Columbia, Vancouver, BC V5Z 1M9, Canada; ytwang@brain.ubc.ca (Y.W.)
5 Campbell Research Institute, Centre for Addiction and Mental Health, Toronto, ON M5T 1R8, Canada.
6 Department of Psychiatry, University of Toronto, Toronto, ON, Canada
* Correspondence: Mxdong64@qdu.edu.cn (M.D.); lisp@pku.edu.cn (S.L.)
† These authors contributed equally to this work.

Received: 10 April 2019; Accepted: 08 May 2019; Published: date

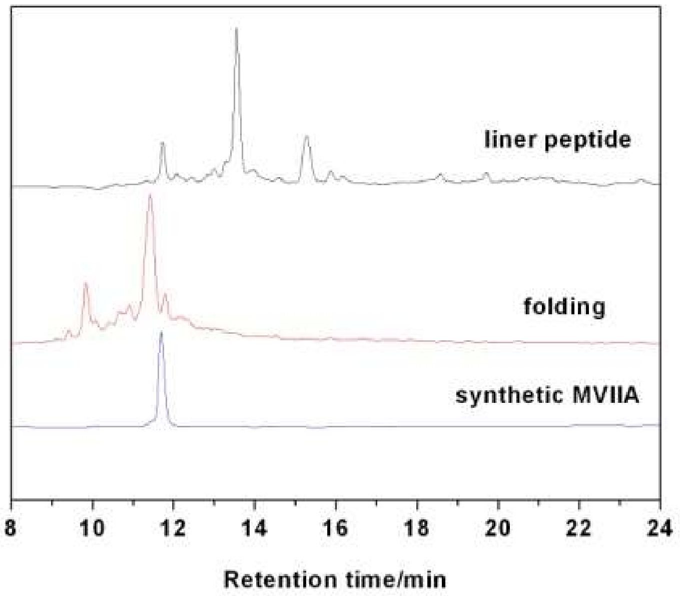

Figure S1. HPLC analyses of one-step folding products of linear MVIIA. Traces from top to bottom: (a) the linear peptide; (b) one-step oxidized products; (c) the purified product of MVIIA. Samples were applied to a Kromasil C18 column (5 μ m, 4.6 mm × 250 mm) and eluted with a linear gradient of 5–10% B for 0–1 min; 10–50% B (B is acetonitrile containing 0.1% TFA) for 1–25 min. Absorbance was monitored at 214 nm. The flow rate was 1.0 mL/min.

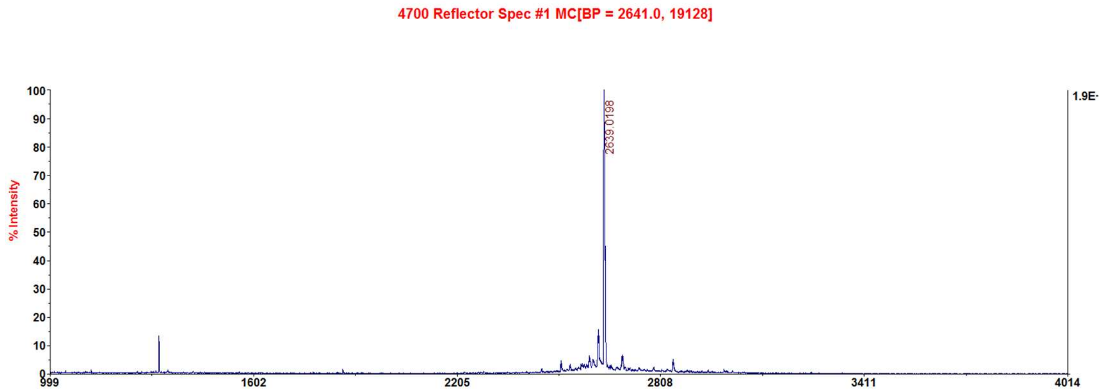

Figure S2. Electrospray ionization mass spectrometry (ESI-MS) data for MVIIA

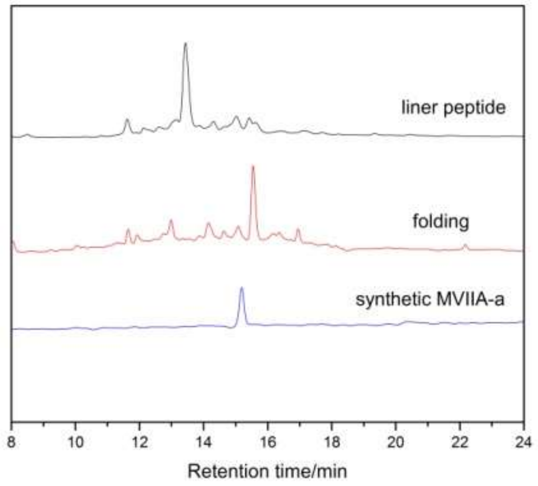

Figure S3. HPLC analyses of one-step folding products of linear MVIIA-a. Traces from top to bottom: (a) the linear peptide; (b) one-step oxidized products; (c) the purified product of MVIIA-a. Samples were applied to a Kromasil C18 column (5 μ m, 4.6 mm × 250 mm) and eluted with a linear gradient of 5–10% B for 0–1 min; 10–50% B (B is acetonitrile containing 0.1% TFA) for 1–25 min. Absorbance was monitored at 214 nm. The flow rate was 1.0 mL/min.

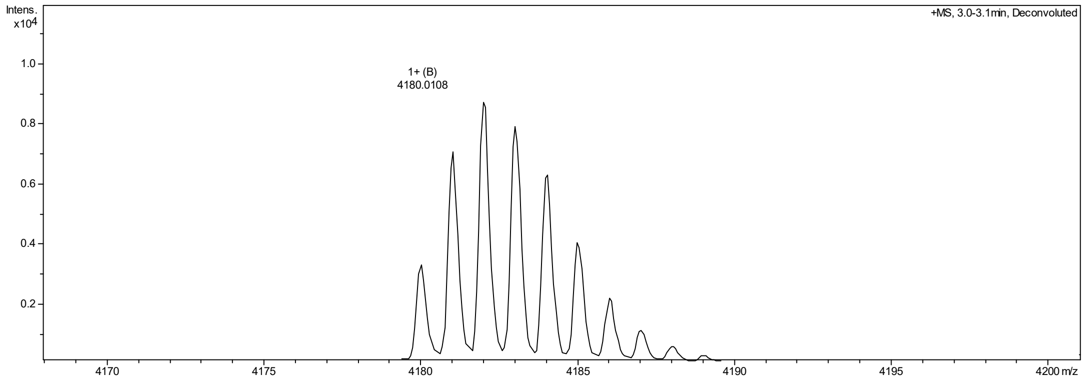

Figure S4. Electrospray ionization mass spectrometry (ESI-MS) data for MVIIA-a

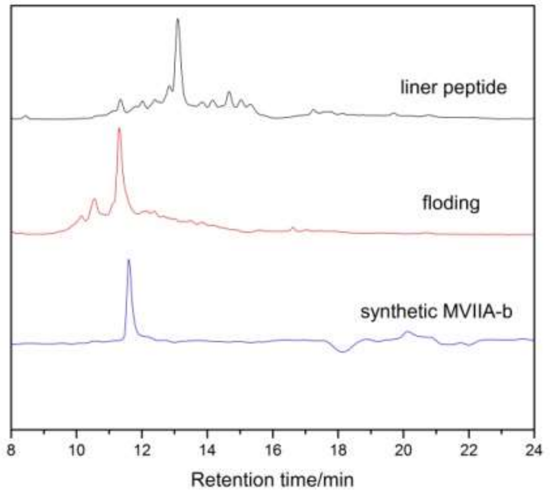

Figure S5. HPLC analyses of one-step folding products of linear MVIIA-b. Traces from top to bottom: (a) the linear peptide; (b) one-step oxidized products; (c) the purified product of MVIIA-b. Samples were applied to a Kromasil C18 column (5 μ m, 4.6 mm × 250 mm) and eluted with a linear gradient of 5–10% B for 0–1 min; 10–50% B (B is acetonitrile containing 0.1% TFA) for 1–25 min. Absorbance was monitored at 214 nm. The flow rate was 1.0 mL/min.

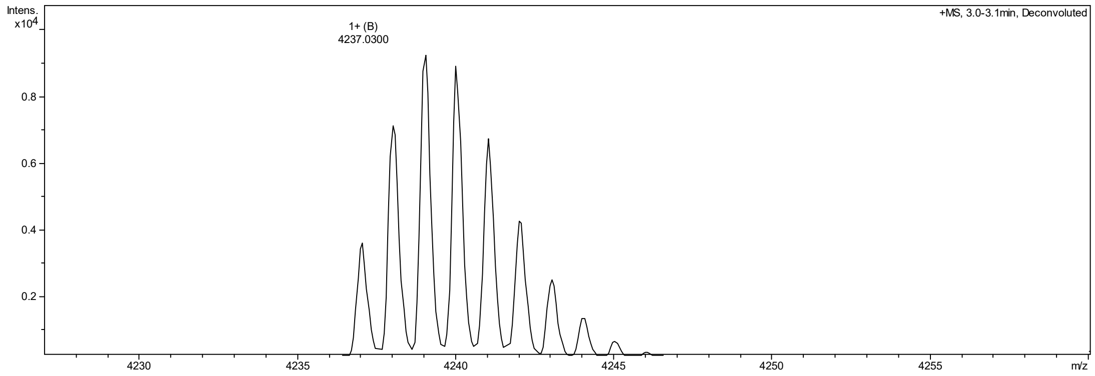

Figure S6. Electrospray ionization mass spectrometry (ESI-MS) data for MVIIA-b

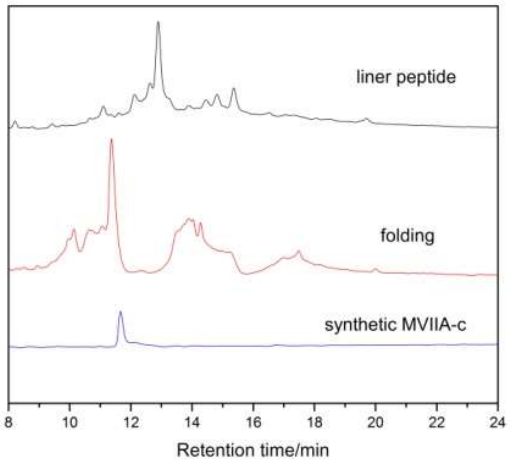

Figure S7. HPLC analyses of one-step folding products of linear MVIIA-c. Traces from top to bottom: (a) the linear peptide; (b) one-step oxidized products; (c) the purified product of MVIIA-c. Samples were applied to a Kromasil C18 column (5 μ m, 4.6 mm × 250 mm) and eluted with a linear gradient of 5–10% B for 0–1 min; 10–50% B (B is acetonitrile containing 0.1% TFA) for 1–25 min. Absorbance was monitored at 214 nm. The flow rate was 1.0 mL/min.

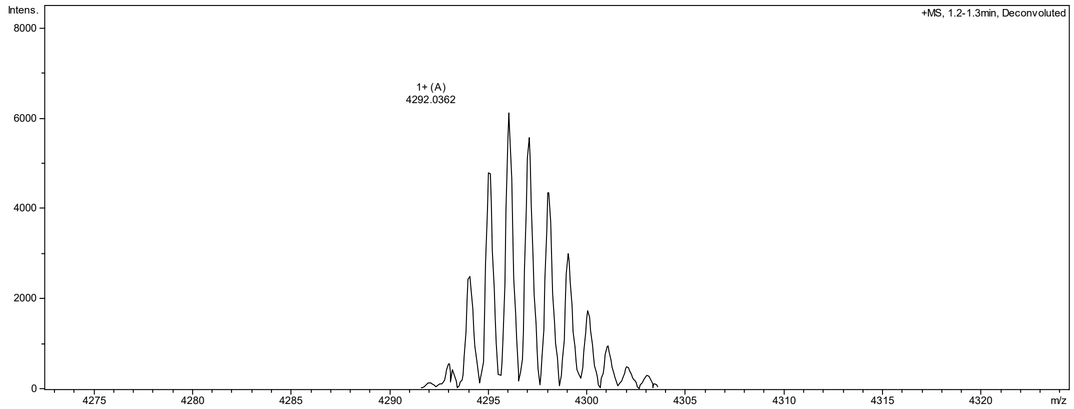

Figure S8. Electrospray ionization mass spectrometry (ESI-MS) data for MVIIA-c

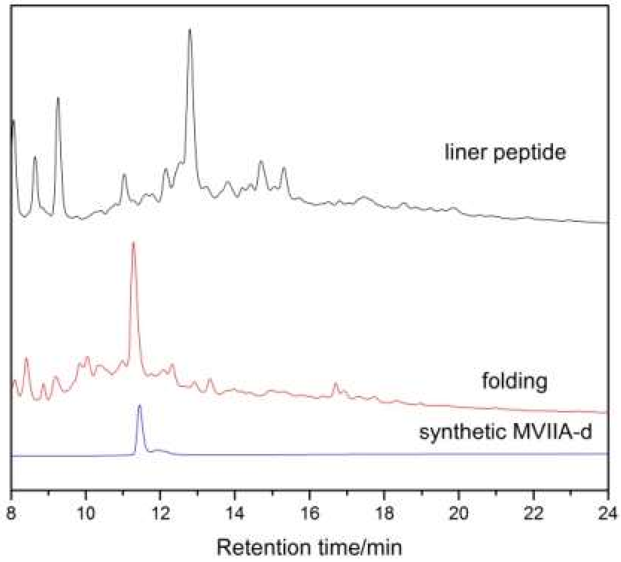

Figure S9. HPLC analyses of one-step folding products of linear MVIIA-d. Traces from top to bottom: (a) the linear peptide; (b) one-step oxidized products; (c) the purified product of MVIIA-d. Samples were applied to a Kromasil C18 column (5 μ m, 4.6 mm × 250 mm) and eluted with a linear gradient of 5–10% B for 0–1 min; 10–50% B (B is acetonitrile containing 0.1% TFA) for 1–25 min. Absorbance was monitored at 214 nm. The flow rate was 1.0 mL/min.

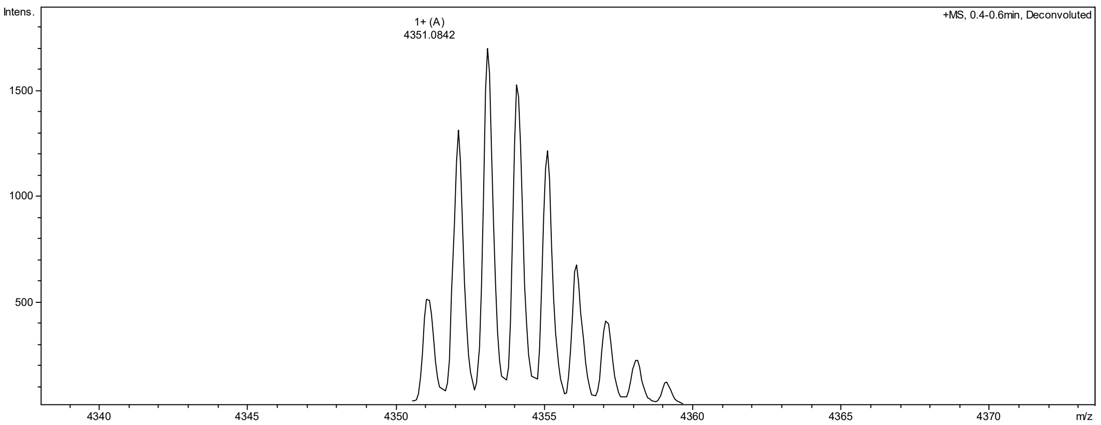

Figure S10. Electrospray ionization mass spectrometry (ESI-MS) data for MVIIA-d
